# CREATION AND EVALUATION OF A VIDEO SERIES SHARING A PALLIATIVE APPROACH TO CARE IN CANADIAN LONG-TERM CARE HOMES

**DOI:** 10.1093/geroni/igae098.1893

**Published:** 2024-12-31

**Authors:** Abigail Wickson-Griffiths, Paulette Hunter, Susan Tupper, Allison Cammer, Sharon Kaasalainen, Tamara Sussman

**Affiliations:** University of Regina, Regina, Saskatchewan, Canada; University of Saskatchewan, Saskatoon, Saskatchewan, Canada; Saskatchewan Health Authority, Saskatoon, Saskatchewan, Canada; University of Saskatchewan, Saskatoon, Saskatchewan, Canada; McMaster University, Hamilton, Ontario, Canada; McGill University, Montreal, Quebec, Canada

## Abstract

Video-assisted patient education is a favored format for accessing healthcare information. Based on a consultative process, the purpose of this project was to create and evaluate a video education resource to support communication between staff and family caregivers in Canadian long-term care (LTC) homes when introducing a palliative approach to care. The development of the resource proceeded in four stages: consulting with lived experience advisors; refining the concept; piloting the concept; and finalizing the resource. The concept included a voice-over-infographic introduction plus a four-part video series highlighting fundamental aspects of a palliative approach in LTC and featuring personal narratives by a resident, family caregiver, employee, and manager. During pilot research, family caregivers and LTC staff (n=16) participated in focus group discussions and surveys to assess the acceptability of the resource and its impact on knowledge acquisition. They also considered potential uses of the resource. After reviewing the introduction and a full video, as well as the concepts for the three remaining videos, the average acceptability of the video series across fourteen 5-point Likert-type items was 4.49 (SD=0.40) for family caregivers and 4.66 (SD=0.35) for LTC staff. The average positive change in palliative care knowledge on a 100-point scale was 6.94 (SD=10.56) for family caregivers and 12.5 (SD=10.76) for LTC staff. Overall, this work contributes a resource with high content and format acceptability, and good potential for a positive impact on knowledge about a palliative approach. It offers useful considerations for introducing the resource to staff and family caregivers.

